# Diabetes, sarcopenia and chronic kidney disease; the Screening for CKD among Older People across Europe (SCOPE) study

**DOI:** 10.1186/s12877-022-02916-9

**Published:** 2022-03-28

**Authors:** Francesc Formiga, Rafael Moreno-González, Andrea Corsonello, Axel Carlsson, Johan Ärnlöv, Francesco Mattace-Raso, Tomasz Kostka, Christian Weingart, Regina Roller-Wirnsberger, Lisanne Tap, Agnieszka Guligowska, Cornel Sieber, Gerhard Wirnsberger, Rada Artzi-Medvedik, Ilan Yehoshua, Cinzia Giuli, Fabrizia Lattanzio, Xavier Corbella, SCOPE investigators, Francesc Formiga, Rafael Moreno-González, Xavier Corbella, Yurema Martínez, Carolina Polo, Josep Maria Cruzado, Andrea Corsonello, Silvia Bustacchini, Silvia Bolognini, Paola D’Ascoli, Raffaella Moresi, Giuseppina Di Stefano, Cinzia Giammarchi, Anna Rita Bonfigli, Roberta Galeazzi, Federica Lenci, Stefano Della Bella, Enrico Bordoni, Mauro Provinciali, Robertina Giacconi, Cinzia Giuli, Demetrio Postacchini, Sabrina Garasto, Annalisa Cozza, Romano Firmani, Moreno Nacciariti, Mirko Di Rosa, Paolo Fabbietti, Johan Ärnlöv, Axel Carlsson, Tobias Feldreich, Francesco Mattace-Raso, Lisanne Tap, Gijsbertus Ziere, Jeannette Goudzwaard, Tomasz Kostka, Agnieszka Guligowska, Łukasz Kroc, Bartłomiej K Sołtysik, Katarzyna Smyj, Elizaveta Fife, Joanna Kostka, Małgorzata Pigłowska, Agnieszka Wójcik, Zuzanna Chrząstek, Natalia Sosowska, Anna Telążka, Christian Weingart, Ellen Freiberger, Cornel Sieber, Gerhard Hubert Wirnsberger, Regina Elisabeth Roller-Wirnsberger, Carolin Herzog, Sonja Lindner, Rada Artzi-Medvedik, Yehudit Melzer, Mark Clarfield, Itshak Melzer, Ilan Yehoshua, Pedro Gil Gregorio, Sara Laínez Martínez, Monica González Alonso, Jose A. Herrero Calvo, Fernando Tornero Molina, Lara Guardado Fuentes, Pamela Carrillo García, María Mombiedro Pérez, Roberto Bernabei, Christophe Bula, Hermann Haller, Carmine Zoccali, Kitty Jager, Wim Van Biesen, Paul E. Stevens

**Affiliations:** 1 Geriatric Unit, Internal Medicine Department, Systemic Diseases, Ageing Group, Cardiovascular, Respiratory, Systemic Diseases, Cellular Aging Program, Institut dInvestigació Biomèdica de Bellvitge IDIBELL, Hospital Universitari de Bellvitge, LHospitalet de Llobregat, Barcelona, Spain; 2 Italian National Research Center On Aging (IRCCS INRCA), Ancona, Fermo and Cosenza, Italy; 3 Department of Neurobiology, Care Sciences and Society, Division of Family Medicine and Primary Care, Karolinska Institute, Huddinge, Sweden; 4 Academic Primary Healthcare Centre, Stockholm, Sweden; 5 Department of Internal Medicine, Section of Geriatric Medicine, Erasmus MC, University Medical Center Rotterdam, Rotterdam, The Netherlands; 6 Department of Geriatrics, Healthy Ageing Research Centre, Medical University of Lodz, Lodz, Poland; 7 Department of Internal Medicine-Geriatrics, Institute for Biomedicine of Aging (IBA), Krankenhaus Barmherzige Brüder, Regensburg, Germany; 8 Department of Internal Medicine, Medical University of Graz, Graz, Austria; 9 Department of Physical Therapy, Recanati School for Community Health Professions at the Faculty of Health Sciences, Ben-Gurion University of the Negev, Beer-sheva, Israel; 10 Maccabi Health Organization, Negev District, Jaffa, Israel

**Keywords:** Sarcopenia, Diabetes, Elderly, Chronic kidney disease, Renal failure

## Abstract

**Background:**

Sarcopenia may be more present in older adults with diabetes (DM). Accordingly, we evaluated the prevalence of sarcopenia and its associated risk factors among community-dwelling older adults with DM.

**Methods:**

A cross-sectional analysis of older people living in the community was carried out. Participants (aged 75 years and more) came from an European multicenter prospective cohort (SCOPE study). Global geriatric assessment including short physical performance battery, handgrip strength test and bioelectrical impedance analysis was performed. Sarcopenia was defined by the updated criteria of the European Working Group on Sarcopenia in Older People (EWGSOP2). Estimated glomerular filtration rate (eGFR) was calculated using Berlin Initiative Study (BIS) to define the stages of chronic kidney disease (CKD). Previous known DM was defined as physician-diagnosed DM registered in the patient's medical record or the use of DM-related medications. Hemoglobin A1c levels and specific DM therapies administered were collected. Time elapsed from the first diagnosis of DM was not collected and, therefore, was not included in the analyses.

**Results:**

A total of 1,420 subjects were evaluated with a median age of 79.0 (6.0) years, of which 804 (56.6%) were women and 615 (43.3%) men; 315 (22.2%) participants had prior DM diagnosis, with a median age of 80.0 (6.0), 146 (46.3%) were women. Using EWGSOP2 definition, 150 (10.6%) participants in the SCOPE study met diagnostic criteria for sarcopenia. Participants without diabetes had more often normal results in the 3 sarcopenia components than participants with diabetes [887 (80.31%) vs. 227 (72.1%), *p* = 0.002], highlighting higher percentages of severe sarcopenia in participants with diabetes [27 (8.6%) vs. 58 (5.2%), *p* = 0.028]. Confirmed or severe sarcopenia was detected in 41 (13%) participants with diabetes and 109 (9.8%) participants without diabetes (*p* = 0.108). According to BIS equation, sarcopenia was not significantly more prevalent in the more advanced stages of CKD (*p* = 0.845). In multivariate analyses, older age (odds ratios [OR], 1.17; 95% confidence interval [CI], 1.08–1.27), and lower body mass index (OR, 0.79; 95% CI, 0.71–0.89 were associated with the presence of sarcopenia.

**Conclusions:**

One tenth of all older community-dwelling subjects have sarcopenia. Older age and being thinner, but not worse renal function, were associated with higher prevalence of sarcopenia in older older adults with diabetes.

## Introduction

Sarcopenia is a muscle disease common among adults of older age defined by low levels measured by three parameters as indicator of severity: muscle strength, muscle quantity/quality and physical performance [1].

A bidirectional association between diabetes and sarcopenia, particularly when complications of diabetes exist has been reported [2]. Thus, a close interrelationship between diabetes mellitus (DM) and sarcopenia has been described, in which longer duration of DM (≥ 6 years) and poorer glycaemic control (HbA1c > 8.0%) have been reported to be associated with lesser muscle quality [3–5]. Furthermore, insulin resistance increases with age, and is underpinning several mechanisms in the induction of sarcopenia. In this regard, insulin is an anabolic hormone, which stimulates protein synthesis including those present in the muscles. Thus, defects in insulin signalling can lead to reduced muscle synthesis [5].

Loss of muscular strength, although it may be generalized, has mainly been described in the lower extremities in older people with DM [6]. Accordingly, a clear relationship between lower quadriceps strength and slower gait speed has been observed in elderly Americans with DM [6]. Similar findings were found in Italy, where older patients with DM had lower walking speed than patients without DM, measured both at a short distance of 4 m and at greater than 400 m [7]. This transverse association between DM and sarcopenia has also been proven to exist in longitudinal studies (almost 5 years of follow-up) confirming a loss in walking speed in people with DM with respect to elderly subjects of the same age without diabetes [8], although no differences in grip strength were observed.

In patients with chronic kidney disease (CKD), the loss of muscle mass is much more intensive and the first signs of sarcopenia are observed in younger patients than expected [9]. Sarcopenia is more common in patients who transit into the most advanced stages of CKD [10]. In a previous SCOPE study using the European Working Group on Sarcopenia in Older People 2 (EWGSOP2) definition, we reported a 10.6% percentage of sarcopenia. In this study, we confirmed that sarcopenia was more prevalent in participants who showed more advanced stages of CKD, according to BIS equation for estimating glomerular filtration rate (eFGR) (9.6% of sarcopenia in patients showing CKD stages 1 and 2 and 13.9% in CKD stages 3a, 3b and 4, *p* = 0.042). Furthermore, a higher prevalence of sarcopenia depending on the severity levels of albuminuria was also documented: 9.3% in normo-albuminuric, 13.2% in micro-albuminuric and 16.8% in macro-albuminuric participants (*p* = 0.019) [11].

Therefore, since micro and macroalbuminuriaare are frequent conditions in elderly patients, it is important to assess if sarcopenia is present, an issue rarely evaluated in routine clinical practice, with even fewer studies performed in Caucasian people [11–14].

In this respect, our first objective was to assess if older community-dwelling people with DM have more sarcopenia than those without DM. Secondly, we evaluated the clinical differences between sarcopenic and non-sarcopenic DM patients. Finally, potential differences in sarcopenia percentages among patients with diabetes were also assessed according to basal eGFR.

## Methods

### Study design and participants

This cross-sectional study used data from the SCOPE study (European Grant Agreement no. 436849), a multicenter 2-year prospective cohort study involving patients older than 75 years attended at geriatric and nephrology outpatient services from participating institutions in Austria, Germany, Israel, Italy, the Netherlands, Poland and Spain. Methods of the SCOPE study have been extensively described elsewhere [11, 15]. Briefly, exclusion criteria were: a) Age < 75 years; b) End-stage renal disease (ESRD) (eGFR < 15 ml/min/1.73 m^2^) or dialysis at the time of enrollment; c) History of solid organ or bone marrow transplantation; d) Active malignancy within 24 months prior to screening or metastatic cancer; e) Life expectancy less than 6 months; f) Severe cognitive impairment (Mini Mental State Examination < 10); g) Any medical (ie. implanted cardioverter-defibrillator or pacemaker) or other reason (e.g. known or suspected inability of the patient to comply with the protocol procedure) in the judgement of the investigators, that the patient is unsuitable for the study; and h) Unwilling to provide consent and those who cannot be followed-up. After obtaining written informed consent, all participants underwent an extensive baseline visit including routine laboratory analysis and comprehensive geriatric assessment (CGA). The baseline visit was followed by follow-up visits at 12 and 24 months with intermediate phone contacts at 6 and 18 months. Only baseline data were used in the present study [11, 15].

Overall, 2,461 participants were initially enrolled in the study. Of them, 204 participants with missing serum creatinine and/or urinary albumin-to-creatinine ratio (ACR) were excluded, thus leaving a sample of 2,257 participants to be included in the initial analysis. For the aim of the present study, only those participants in whom sarcopenia could be assessed in its three components (muscle strength, muscle mass and physical performance) were considered. The variables muscle strength assessed by grip strength; muscle mass by bioelectrical impedance analysis (BIA); and physical performance by the Short Physical Performance Battery (SPPB) were available for 2,138 (94.7%), 1,462 (64.8%) and 2,256 (99.9%) participants, respectively. Participants with missing data mainly included those physically unable or unsteady, those presenting arthralgia or arthritis, those with an implanted cardioverter-defibrillator or pacemaker, or those not assessed due to any other safety reason in the judgement of the study investigators. In total, 1,420 participants were finally included [11, 15].

Anthropometric measures were collected and body mass index (BMI) was calculated by dividing body weight by height squared (kg/m^2^) [16]. Cognitive function was assessed by the Mini Mental State Examination (MMSE) [17]; depressive symptoms were assessed by the Geriatric Depression Scale (GDS) in its short form [18]; the ability to perform activities of daily living (ADL) [19] and instrumental activities of daily living (IADL) [20] was also assessed. The Cumulative Illness Rating Scale for geriatrics (CIRS-G) [21] was administered to account for comorbidity burden. There were no statistically significant differences between the included and excluded participants (either missing serum creatinine or ACR, or any measurement required for sarcopenia full assessment in its three components) in terms of age, gender, living alone rate, education years, ADL score, MMSE score, number of chronic medications and serum creatinine levels, although higher IADL score, GDS score and CIRS-G total score.

### Assessment of sarcopenia

Following the revised EWGSOP2 criteria for an operational definition of sarcopenia [1], all three components: muscle strength, muscle mass, and physical performance were assessed. Probable sarcopenia was identified when low muscle strength was present. Diagnosis of sarcopenia was confirmed when low muscle strength and low muscle mass were both present, and criteria for severe sarcopenia were met when sarcopenia was concurred with low physical performance. The SARC-F questionnaire was not applied as we did the initial screening.

Muscle strength was assessed by the handgrip strength test [22], using a hydraulic grip strength dynamometer (Model J00105 JAMAR Hydraulic Hand, Lafayette Instrument Company, USA). Participants were encouraged to squeeze as hard as they could, 3 attempts were allowed for each hand alternating sides and the maximum measurement was registered. EWGSOP2 recommended cut-off points for low muscle strength were used, < 27 kg for men and < 16 kg for women.

Body composition in terms of fat and fat-free mass was assessed by BIA using the AKERN BIA 101 New Edition 50 kHz monofrequency device (AKERN SRL, Florence, Italy). Appendicular skeletal muscle mass (ASM) was estimated using the Sergi et al. equation [23], a cross-validated equation for standardization specifically derived from older European populations, as recommended by the EWGSOP2 consensus. A decision was made to apply no adjustment for body size to ASM measures, as also contemplated in the consensus. Following the EWGSOP2 cut-off points, low muscle mass was defined by an ASM < 20.0 kg for men and < 15 kg for women.

Physical performance was assessed by the SPPB, a composite test consisting of a balance test (ability to stand for 10 s with feet close together side by side, then in semi-tandem and then in full-tandem positions), a gait speed assessment (usual time to walk 4 m), and a chair stand test (time to raise from a chair and return to the seated position 5 times without using arms) [24]. A score from 0 to 4 was assigned to each test, thus summing up to a maximum total score of 12. As suggested by the EWGSOP2 consensus, a total score of ≤ 8 was considered to indicate low physical performance.

Men and women were evaluated together, applying the different diagnostic criteria in each case [11].

### Assessment of kidney function

Serum creatinine was measured at local level by standard methods. Creatinine-based eGFR was calculated using the Berlin Initiative Study equation (BIS) [25]: 3736 × creatinine^−0.87^ × age^−0.95^ × 0.82(if female). Categories of CKD were defined according to the Kidney Disease Improving Global Outcomes (KDIGO) guidelines [26]. Moreover, CKD categories were later combined into two groups: eGFR ≥ 60 ml/min/1.73 m^2^ (categories 1 and 2) and eGFR < 60 ml/min/1.73 m^2^ (categories 3a, 3b and 4). Albumin in urine was detected by urine spot analysis and expressed as mg albumin per gram urine (mg/g), and albumin-to-creatinine ratio (ACR) was calculated and reported as mg albumin per gram creatinine (mg/g). Severity categories of albuminuria were also defined according to the KDIGO guidelines: normo-albuminuria was defined as ACR < 30 mg/g, micro-albuminuria as ACR 30–300 mg/g and macro-albuminuria as ACR > 300 mg/g.

### Assessment of diabetes

Previous known DM was defined as physician-diagnosed DM registered in the patient's medical record or the use of DM-related medications. Hemoglobin A1c levels DM and specific therapies administered from DM diagnosis were collected. Time elapsed from the first diagnosis of DM was not collected and, therefore, was not included in the analyses.

### Ethics

All patients or their representatives signed an informed consent before being recruited for the study. Confidential information of the patients was protected according to national normative. The study protocol was approved by ethics committees at all participating institutions, and complies with the Declaration of Helsinki and Good Clinical Practice Guidelines.

### Statistical analysis

All continuous variables were checked for normality by the Kolmogorov–Smirnov test. They were all non-normally distributed, so they were expressed as median with interquartile difference. Categorical variables were expressed as number and percentage.

First, anthropometric measurements, BIA parameters, and sarcopenia components and categories were compared based on the presence or absence of DM.

Second, only for participants with diabetes, baseline characteristics, clinical management and in-hospital clinical course were compared based on the presence or absence of sarcopenia.

The differences between categorical variables was analyzed by the Chi-square test, with the correction of continuity when indicated. The differences between quantitative variables, according to sarcopenia categories, was analyzed by Mann-Witney test.

In order to obtain an estimate of the association with sarcopenia in participants with diabetes, a logistic regression analyses was performed. The model first model were was adjusted for age and gender, and the fully adjusted model model was adjusted fot age, gender, education level, hypertension, IADL, MMSE and BMI.

Statistical significance was set at *p* < 0.05. All statistical analyses were performed with SPSS version 24 (SPSS Inc., Chicago, IL, USA) and MedCalc (JMP® statistics software, USA).

## Results

A total of 1,420 subjects were evaluated with a median age of 79.0 (6.0) years, of which 804 (56.6%) were women and 615 (43.3%) men.

### Participants with diabetes

315 (22.2%) participants had prior DM diagnosis, with a median age of 80.0 (6.0), 146 (46.3%) were women, 73 (23%) were living alone and median of educational years was 10.0 (5.0).

Overall median BMI was 27.0 kg/m^2^ (5.6). BMI values were higher in participants with diabetesthan in those without diabetes (28.6 (5.0) vs. 26.5 (5.3)); *p* < 0.001. Fat mass percentage (FM%) (overall median 31.0 (12.0)) was also higher in participants with diabetes (32.0 (13.0) vs. 30.6 (12.0); *p* = 0.008).

Median grip strength was 24.0 (13.0) kg in the total participants, and there were not differences between both groups (24.0 (13.0) vs. 24.0 (13.0); *p* = 0.655). Notwithstanding, a higher percentage of participants with diabetes showed low muscle strength (27.9% vs. 19.7%; *p* = 0.002).

Median ASM as derived from BIA was 18.1 (6.5) kg (19.5 (6.6) vs. 17.7 (6.2); *p* < 0.001) and following the EWGSOP2 recommended cut-off values, there was a lower percentage of participants with diabetes with low muscle mass (25.7% vs. 34.6%; *p* = 0.003). Regarding the evaluation of SPPB, overall median score was 10.0 (3.0), and considering the recommended cut-off value of ≤ 8 points, participants with diabetes had lower physical performance (44.4% vs. 27.1%; *p* < 0.001).

Table 1 shows anthropometric measurements, BIA parameters, sarcopenia components and sarcopenia categories, stratified by prior diabetes diagnosis. Participants without diabetes showed more often normal results in the 3 components of sarcopenia than participants with diabetes (80.3% vs 72.1%, *p* = 0.002); moreover, there were higher percentages of severe sarcopenia in participants with diabetes (5.2% vs 8.6%, *p* = 0.028). Confirmed or severe sarcopenia was detected in 109 (9.8%) participants without diabetes and in 41 (13%) participants with diabetes (*p* = 0.108). Table 2 shows differences between the 315 diabetic participants with respect to sarcopenia (confirmed or severe sarcopenia) or no sarcopenia (not sarcopenic or probable sarcopenia) status (41 vs. 274 patients, respectively).

**Table 1:** Anthropometric measurements, BIA parameters, sarcopenia components and sarcopenia categories, stratified by prior diabetes diagnosis

	**Total** **(*n* = 1420)**	**No diabetes** **(*n* = 1105)**	**Diabetes (*n* = 315)**	**p-value**
**Anthropometric measurements**				
height, cm	162.0 (14.0)	162.0 (13.0)	164.5 (13.0)	0.056
weight, kg	**72.0 (20.0)**	**71.0 (19.0)**	**77.5 (19.0)**	** < 0.001**
BMI, kg/m^2^	**27.0 (5.6)**	**26.5 (5.3)**	**28.6 (5.0)**	** < 0.001**
BIA parameters				
FFM, percentage	**69.0 (12.0)**	**69.3 (12.0)**	**68.0 (13.0)**	**0.008**
FFM, kg	**48.5 (15.0)**	**47.5 (14.0)**	**51.9 (16.0)**	** < 0.001**
FFMI, kg/m^2^	**18.5 (3.0)**	**18.3 (3.0)**	**19.3 (3.0)**	** < 0.001**
FM, percentage	**31.0 (12.0)**	**30.6 (12.0)**	**32.0 (13.0)**	**0.008**
FM, kg	**21.9 (11.9)**	**21.2 (13.4)**	**24.3 (11.8)**	** < 0.001**
FMI, kg/m^2^	**8.3 (4.5)**	**8.0 (4.5)**	**9.3 (5.1)**	** < 0.001**
ASM, percentage	0.25 (0.04)	0.25 (0.05)	0.25 (0.05)	0.831
ASM, kg	**18.1 (6.5)**	**17.7 (6.2)**	**19.5 (6.6)**	** < 0.001**
ASMI, kg/m^2^	**6.9 (1.3)**	**6.8 (1.4)**	**7.3 (1.4)**	** < 0.001**
Grip strength, kg	24.0 (13.0)	24.0 (13.0)	24.0 (13.0)	0.655
SPPB score	**10.0 (3.0)**	**10.0 (3.0)**	**9.0 (5.0)**	** < 0.001**
**Sarcopenia components**				
Low muscle strength	**306 (21.5)**	**218 (19.7)**	**88 (27.9)**	**0.002**
Low muscle mass	**463 (32.6)**	**382 (34.6)**	**81 (25.7)**	**0.003**
Low physical performance	**440 (31.0)**	**300 (27.1)**	**140 (44.4)**	** < 0.001**
**Sarcopenia categories**				
No sarcopenia	**1114 (78.5)**	**887 (80.3)**	**227 (72.1)**	**0.002**
Probable sarcopenia	**156 (11.0)**	**109 (9.9)**	**47 (14.9)**	**0.011**
Confirmed sarcopenia	65 (4.6)	51 (4.6)	14 (4.4)	0.898
Severe sarcopenia	**85 (6.0)**	**58 (5.2)**	**27 (8.6)**	**0.028**

Continuous variables are expressed as median and interquartile difference. Abbreviations; *ASM* appendicular skeletal muscle mass, *ASMI* appendicular skeletal muscle mass index, *BIA* bioelectrical impedance analysis, *BMI* body mass index, *FFM* fat-free mass, *FFMI* fat-free mass index, *FM* fat mass, *FMI* fat mass index, *SSPB* short physical performance battery

**Table 2:** Differences between sarcopenic and non-sarcopenic participants with diabetes

	**Participants with diabetes (*n* = 315)**	**Non sarcopenic (*n* = 274)**	**Sarcopenic (*n* = 41)**	**p-value**
**Age, years**	**80.0 (6.0)**	**79.0 (6.0)**	**83.0 (7.0)**	** < 0.001**
Women, n (%)	146 (46.3)	128 (46.7)	18 (43.9)	0.736
Living alone, n (%)	73 (23.2)	63 (23.0)	10 (24.4)	0.843
**Education, years**	**10.0 (5.0)**	**10.0 (5.0)**	**8.0 (6.5)**	**0.013**
**Comorbidities**				
** Hypertension, n (%)**	**276 (87.6)**	**245 (89.4)**	**31 (75.6)**	**0.012**
Stroke, n (%)	26 (8.3)	22 (8.0)	4 (9.8)	0.708
Hip fracture, n (%)	14 (4.4)	11 (4.0)	3 (7.3)	0.339
Osteoporosis, n (%)	74 (23.5)	61 (22.3)	13 (31.7)	0.183
Parkinson, n (%)	12 (3.8)	9 (3.3)	3 (7.3)	0.208
At least 1 BADL dependent (intensive assistance or dependent), n (%)	20 (6.3)	16 (5.8)	4 (9.8)	0.337
**IADL score**	**4.0 (12.0)**	**4.0 (11.0)**	**9.0 (16.0)**	** < 0.001**
**At least 1 IADL dependent (intensive assistance or dependent), n (%)**	**160 (50.8)**	**131 (47.8)**	**29 (70.7)**	**0.006**
MMSE adjusted score	28.0 (2.7)	28.0 (2.4)	27.0 (5.2)	0.117
**MMSE adjusted < 24, n (%)**	**33 (10.5)**	**23 (8.4)**	**10 (24.4)**	**0.002**
GDS score	2.0 (2.3)	2.0 (3.0)	2.0 (3.0)	0.471
GDS > 5, n (%)	36 (11.5)	32 (11.7)	4 (9.8)	0.713
CIRS-G total score	10.0 (7.0)	10.0 (7.0)	10.0 (7.0)	0.721
CIRS-G severity index	1.6 (0.6)	1.6 (0.5)	1.6 (0.5)	0.793
Number of current medications	9.0 (4.0)	8.0 (4.3)	9.0 (4.0)	0.532
Creatinine, mg/dl	1.1 (0.6)	1.1 (0.7)	1.1 (0.8)	0.832
BIS eGFR, ml/min/1.73 m^2^	48.3 (22.2)	48.6 (22.0)	46.2 (29.0)	0.877
≥ 90	2 (0.6)	2 (0.7)	0 (0.0)	0.845
60–89	74 (23.5)	62 (22.6)	12 (29.3)	
45–59	106 (33.7)	94 (34.3)	12 (29.3)	
30–44	99 (31.4)	87 (31.8)	12 (29.3)	
< 30	34 (10.8)	29 (10.6)	5 (12.2)	
≥ 60	76 (24.1)	64 (23.4)	12 (29.3)	0.409
< 60	239 (75.9)	210 (76.6)	29 (70.7)	
ACR, mg/g	21.0 (80.0)	19.4 (79.0)	35.9 (213.0)	0.091
< 30	180 (57.1)	162 (59.1)	18 (43.9)	0.132
30–300	91 (28.9)	77 (28.1)	14 (34.1)	
> 300	44 (14.0)	35 (12.8)	9 (22.0)	
Albumin, g/dl	4.0 (1.0)	4.0 (1.2)	4.0 (0.6)	0.879
Fasting plasma Glucose, mg/dl	106.0 (133.0)	106.0 (132.0)	105.0 (140.0)	0.809
Hemoglobin A1c %	6.5 (1.6)	6.5 (1.6)	6.4 (1.2)	0.773
Cholesterol, mg/dl	155.0 (193.0)	158.0 (194.0)	135.0 (178.0)	0.542
Triglycerides, mg/dl	94.0 (141.0)	96.0 (142.0)	79.5 (113.0)	0.350
Vitamin D, ng/mL	26.1 (27.0)	25.7 (26.0)	32.5 (34.0)	0.519
DM therapy				
Insulin, n (%)	96 (30.6)	84 (30.7)	12 (30.0)	0.933
Metformin, n (%)	172 (54.8)	150 (54.7)	22 (55.0)	0.976
Sulfonylureas, n (%)	67 (21.3)	60 (21.9)	7 (17.5)	0.526
DPP4-I, n (%)	14 (4.5)	10 (3.6)	4 (10.0)	0.069
SGLT2, n (%)	2 (0.6)	2 (0.7)	0 (0.0)	0.588
GLP-1 RAs, n (%)	4 (1.3)	4 (1.5)	0 (0.0)	0.442
Anthropometric measurements				
** Height, cm**	**164.5 (23.0)**	**165.1 (15.0)**	**158.5 (11.0)**	** < 0.001**
** Weight, kg**	**77.5 (19.0)**	**79.9 (17.0)**	**60.7 (12.0)**	** < 0.001**
** BMI, kg/m**^**2**^	**28.6 (4.0)**	**29.1 (5.1)**	**26.3 (4.6)**	** < 0.001**
BIA parameters				
FFM, percentage	68.0 (12.8)	68.0 (13.8)	69.0 (18.0)	0.071
** FFM, kg**	**51.9 (16.0)**	**53.6 (17.2)**	**42.6 (19.0)**	** < 0.001**
** FFMI, kg/m**^**2**^	**19.3 (3.1)**	**19.6 (3.3)**	**18.1 (2.1)**	** < 0.001**
FM, percentage	32.0 (12.8)	32.0 (13.8)	31.0 (8.1)	0.082
** FM, kg**	**24.3 (11.8)**	**26.0 (12.1)**	**20.0 (7.6)**	** < 0.001**
** FMI, kg/m**^**2**^	**9.3 (5.1)**	**9.5 (5.4)**	**8.1 (3.4)**	**0.002**
ASM, percentage	0.25 (0.05)	0.25 (0.05)	0.25 (0.05)	0.919
** ASM, kg**	**19.5 (6.6)**	**20.6 (6.3)**	**16.4 (4.9)**	** < 0.001**
** ASMI, kg/m**^**2**^	**7.3 (1.4)**	**7.4 (1.5)**	**6.4 (1.3)**	** < 0.001**
Grip strength, kg	24.0 (13.0)	24.0 (13.0)	24.0 (13.0)	0.655
**SPPB score**	**9.0 (5.0)**	**9.0 (5.0)**	**8.0 (5.0)**	**0.025**
SPPB Balance	4.0 (2.0)	4.0 (2.0)	3.0 (2.0)	0.101
SPPB Gait Speed	3.0 (2.0)	3.0 (2.0)	3.0 (2.0)	0.092

Continuous variables are expressed as median and interquartile difference. Abbreviations; *ACR* albumin-to-creatinine ratio, *ADL* activities of daily living, *ASM* appendicular skeletal muscle mass, *ASMI* appendicular skeletal muscle mass index, *BIA* bioelectrical impedance analysis, *BIS* Berlin Initiative Study, *BMI* body mass index, *CIRS-G* cumulative illness rating scale for geriatrics, *CKD-EPI* Chronic Kidney Disease Epidemiological Collaboration, *DM* diabetes mellitus, *eGFR* estimated glomerular filtration rate, *FAS* Full Age Spectrum, *FFM* fat-free mass, *FFMI* fat-free mass index, *FM* fat mass, *FMI* fat mass index, *GDS* geriatric depression scale, *IADL* instrumental activities of daily living, *MMSE* mini mental state examination, *SSPB* short physical performance battery, *DPP4-I* dipeptidyl peptidase-4 inhibitors, *SGLT2* Sodium-glucose cotransporter-2 inhibitors, *GLP-1 RAs* glucagon-like peptide receptor agonists

Among participants with diabetes, those with sarcopenia were older (83.0 vs. 79.0 years; *p* < 0.001), with lesser years of education (8.0 vs. 10.0; *p* = 0.013) and physically were significantly shorter (*p* < 0.001), thinner (*p* < 0.001), and with lower BMI (*p* < 0.001). Among the comorbidities evaluated, DM patients with sarcopenia only differed from non sarcopenic showing a lower proportion of previous diagnosis of hypertension (*p* = 0.012). Moreover, DM patients with sarcopenia had poorer performance in IADL (*p* < 0.001) and lower cognition scores (*p* = 0.002). Regarding DM therapy at the time of basal assessment, there was no difference between participants with or without sarcopenia, either with the use of insulin or with any other oral drug for DM.

### Sarcopenia according to kidney function in participants with diabetes

According to BIS equation (Table 2), sarcopenia was not significantly more prevalent in patients with the most advanced stages of CKD (*p* = 0.845). The distribution of participants according to ACR categories showed higher prevalence rates of sarcopenia with increasing albuminuria categories, although without statistical significance (*p* = 0.132).

Table 3 shows factors identified as associated with predictive sarcopenia in patients with diabetes. In this regard, analysis adjusted by age and gender showed that, in older patients with diabetes, sarcopenia was associated with older age, male gender, more years of education, absence of hypertension, and more IADL dependence or poor cognitive performance (MMSE adjusted < 24) besides lesser BMI. In multivariate analyses, older age (odds ratios [OR], 1.17; 95% confidence interval [CI], 1.08–1.27), and lower body mass index (OR, 0.79; 95% CI, 0.71–0.89 were both associated with sarcopenia in our study population of older adults with diabetes.

**Table 3:** Factors identified as associated with sarcopenia in participants with diabetes

Variable	Age and gender adjusted model OR (95%CI)		Fully adjusted model OR (95%CI)	
**Age, years**	-	-	1.17	1.08–1.27
**Male Gender**	-	-	1.33	0.59–2.99
**Education, years**	0.92	0.85 – 0.99	0.93	0.85–1.01
**Hypertension**	0.34	0.14 – 0.82	0.57	0.21–1.53
**At least 1 IADL dependent (intensive assistance or dependent**	2.29	1.03 – 5.07	2.27	0.96–5.41
**MMSE adjusted < 24**	3.03	1.26 – 7.31	1.65	0.56–4.81
**BMI, kg/m**^**2**^	0.81	0.72 – 0.89	0.79	0.71–0.89
SPPB score	0.94	0.83 – 1.05		

^*^in fully adjusted model we consider all the variables in bold

*IADL* instrumental activities of daily living, *MMSE* mini mental state examination, *BMI* body mass index, *SSPB* short physical performance battery

## Discussion

Main findings from our study were that participants with diabetes had a higher frequency of poor results in the 3 components of sarcopenia than participants without diabetes, thus having a higher proportion of sarcopenia and severe sarcopenia. Furthermore, older age and lower BMI, but not kidney function, were associated with a higher prevalence of sarcopenia in our cohort of older participants with diabetes.

Previously epidemiological studies have reported a wide range of sarcopenia prevalence in patients with diabetes, varying from 7 to 29% in different populations and using different inclusion criteria [27]. The prevalence of sarcopenia in our study was 13% using EWGSOP2 criteria in a cohort of 315 elderly patients with diabetes with a mean age of 80 years. Only few studies in younger aged patients with diabetes are available to compare sarcopenia prevalence using EWGSOP2 criteria. In this regard, a Brazilian study including 242 patients with diabetes (mean age 68.3 years) reported that the prevalence of sarcopenia was more than double when comparing EWGSOP1 (16.9%) and EWGSOP2 (7%) [28]. Another smaller Australian study (87 patients, mean age 71 years), also reported more cases of sarcopenia in patients with diabetes using EWGSOP1 criteria (prevalence of 7%) than with EWGSOP2 (prevalence of 2%) [29].

Evaluating the components of sarcopenia separately, we confirmed some previously reported results in older women (mean age 78.5 years) [8] in whom grip strength was similar among subjects with and without diabetes. However, other studies found that grip strength was lower among those people with known and newly diagnosed diabetes in comparison with those normo-glycaemic [30].

Sarcopenia has previously been reported to be associated with declining renal function in patients with diabetes [12], though in a younger population than the present cohort. Comparatively, the older age of our participants and the fact that a high percentage (up to three quarters) of the SCOPE participants with diabetes had some degree of CKD, may explain why no significant association was found between eGFR and sarcopenia in our cohort of diabetic participants. Besides, the use of creatinine-based eGFR equations may be misleading and could be masking a possible association between CKD and sarcopenia, due to sarcopenic participants (and consequently with low muscle mass) exhibiting lower serum creatinine levels, irrespective of their kidney function. Regarding kidney function it has also been shown that among diabetic patients, sarcopenia was more prevalent in those individuals with albuminuria (ACR ≥ 30 μg/mg) than in those without [13]. The results of the present study are in line with these previous findings, though without statistical significance, probably due to the small numbers of participants in each category of albuminuria.

In our study, analysis adjusted by age and sex showed that in elderly participants with diabetes, sarcopenia is associated with higher age, male gender, higher education, absence of hypertension as a comorbidity, more IADL dependence, poor cognitive performance (MMSE adjusted < 24) and lower BMI. In multivariate analyses older age and lower BMI were both associated with sarcopenia in our transversal study of participants with diabetes. In this regard, age-related decline in exercise capacity is a well-known major factor in the decline of muscle mass and muscle strength in older adults [31]. Therefore, our results may suggest that patients with sarcopenia had a lower BMI due to the loss of muscle mass. The relationship between sarcopenia and cognition in our diabetic participants was not maintained in the multivariate analysis, though it is known that skeletal muscle produces and secretes myokines that regulate brain functions and participate in the muscle-brain endocrine loop [32].

In a study using the Asian Working Group criteria for sarcopenia [31] with 38 sarcopenic subjects, logistic regression analysis showed that older age (OR: 1.182), trunk fat mass (OR: 1.499) and free thyroxine (OR: 1.342) were independent risk factors for sarcopenia. Also lower BMI (OR: 0.365), exercise practice (OR: 0.016), female gender (OR: 0.000), metformin use (OR: 0.159) and greater trunk skeletal muscle mass (OR: 0.395) were protective factors for sarcopenia. In another interesting study, poor glycemic control was associated with low muscle mass in Japanese patients with DM (mean age 69.9 years) [33]. However, the present study could not confirm such differences between both groups in either basal fasting plasma glucose or hemoglobin A1c %.

Indirectly, insulin therapy may improve muscle health in aging subjects [34]. This effect of insulin may explain why no differences in sarcopenia rates were found between insulin-dependent and non-insulin-dependent patients in our study, even though the former study probably included patients with more years of diabetes evolution and complications. Regarding oral pharmacological therapy for DM, it has been reported that dipeptidyl peptidase-4 inhibitors (DPP4-I) use may have a beneficial effect on the prevention of loss of muscle mass and its function compared with sulfonylureas [35]. In our study, we could not demonstrate this effect of DPP4-I (probably due to low number of participants receiving this therapy), nor any significant association between current DM therapy at baseline and sarcopenia.

Our study has several limitations. First, we did not assess neither the “severity” of DM diagnosis (i.e. time from DM onset to first hospital admission, DM-related complications or type and intensity of DM therapies used). Second, we did not distinguish between type 1 and type 2 DM, although it was assumed that the great majority of DM diagnoses in our elderly population aged ≥ 75 years were type 2 DM. Furthermore, causality in the relationship between sarcopenia and diabetes cannot be established in a cross-sectional analysis. The number of patients with diabetes who were diagnosed with sarcopenia was limited and may not have been sufficiently large to detect a significant difference between sarcopenia and kidney function parameters. Finally, the fact that more than one third of the study population was excluded due to missing data could introduce a bias, although assessment of the excluded participants showed no statistically significant differences in main variables of interest.

## Conclusions

Sarcopenia was present in one tenth of our older community-dwelling subjects with diabetes. Older age and lower BMI, but not kidney function, were associated with a higher prevalence of sarcopenia in these older participants with diabetes.

## Data Availability

The data cannot be shared publicly because there was no such approval in the study protocol. The datasets used and analyzed during the study are available from the corresponding author upon reasonable request and subject to ethical approval request.

## Abbreviations

ACR: Albumin-to-creatinine ratio
ADL: Activities of daily living
ASM: Appendicular skeletal muscle mass
ASMI: Appendicular skeletal muscle mass index
AWGS: Asian Working Group for Sarcopenia
BIA: Bioelectrical impedance analysis
BIS: Berlin Initiative Study
BMI: Body mass index
CGA: Comprehensive geriatric assessment
CIRS-G: Cumulative illness rating scale for geriatrics
CKD: Chronic kidney disease
CKD-EPI: Chronic Kidney Disease Epidemiological Collaboration
DXA: Dual-energy X-ray absorptiometry
eGFR: Estimated glomerular filtration rate
ESPEN: European Society of Clinical Nutrition
ESRD: End-stage renal disease
EWGSOP: European Working Group on Sarcopenia in Older People
FAS: Full Age Spectrum
FFM: Fat-free mass
FFMI: Fat-free mass index
FM: Fat mass
FMI: Fat mass index
FNIH: Foundation for the National Institutes of Health
GDS: Geriatric depression scale
GFR: Glomerular filtration rate
IADL: Instrumental activities of daily living
IWGS: International Working Group on Sarcopenia
KDIGO: Kidney Disease Improving Global Outcomes
MDRD: Modification of Diet in Renal Disease
MMSE: Mini mental state examination
SCOPE: Screening for Chronic Kidney Disease among Older People across Europe
SCWD: Society on Sarcopenia, Cachexia and Wasting Disorders
SMM: Skeletal muscle mass
SMMI: Skeletal muscle mass index
SSPB: Short physical performance battery
